# Highly Selective Detection of Paraoxon in Food Based on the Platform of Cu Nanocluster/MnO_2_ Nanosheets

**DOI:** 10.3390/nano12091429

**Published:** 2022-04-22

**Authors:** Shuo Liu, Peng Zhang, Yuming Miao, Chenmin Li, Yu-e Shi, Jinhua Liu, Yun-kai Lv, Zhenguang Wang

**Affiliations:** 1 Key Laboratory of Chemical Biology of Hebei Province, Key Laboratory of Medicinal Chemistry and Molecular Diagnosis, Ministry of Education, College of Chemistry & Environmental Science, Hebei University, Baoding 071002, China; 1768345433@163.com (S.L.); 1060191408@163.com (Y.M.); 2210445091@163.com (C.L.); lvyu163nkai@126.com (Y.-k.L.); 2 Department of Pharmacy, Shenzhen Luohu People’s Hospital, No. 47 Youyi Rd, Luohu, Shenzhen 518001, China; pzhang898018@163.com; 3 Key Laboratory of Flexible Electronics (KLOFE), Institute of Advanced Materials (IAM), Nanjing Tech University, 30 South Puzhu Road, Nanjing 211816, China; iamjhliu@njtech.edu.cn

**Keywords:** organophosphorus pesticides, fluorometry assay, metal nanoclusters, MnO_2_ nanosheets, fluorescence quenching

## Abstract

Selective and sensitive identification of paraoxon residue in agricultural products is greatly significant for food safety but remains a challenging task. Herein, a detection platform was developed by integrating Cu nanoclusters (Cu NCs) with MnO_2_ nanosheets, where the fluorescence of Cu NCs was effectively quenched. Upon introducing butyrylcholinesterase and butyrylcholine into the system, their hydrolysate, thiocholine, leads to the decomposition of the platform through a reaction between the MnO_2_ nanosheets and thiol groups on thiocholine. The electron-rich groups on thiocholine can further promote the fluorescence intensity of Cu NCs through host–guest interactions. Adding paraoxon results in the failure of fluorescence recovery and further promotion, which could be utilized for the quantitative detection of paraoxon, and a limit of detection as low as 0.22 ng/mL can be achieved. The detection platform shows strong tolerance to common interference species, which endows its applications for the detection of paraoxon in vegetables and fruit. These presented results not only open a new door for the functionalization of metal nanoclusters but also offer an inspiring strategy for analytic techniques in nanomedicine and environmental science.

## 1. Introduction

Organophosphate pesticides (OPs) are widely used for pest control in the field of industry, agriculture, and human premises, significantly improving agricultural yields and sanitary conditions [1,2]. OPs kill pests by inhibiting the enzyme activity of acetylcholinesterase (AChE) via the mechanism of phosphorylating AChE. This results in the failure of AChE to catalyze acetylcholine, which further leads to the over-accumulation of acetylcholine and cholinergic toxicity [3,4]. Among them, paraoxon is the most widely used and noxious type of OPs [5,6]. Thus, the selective and sensitive identification of paraoxon residue in agricultural products is greatly significant for food safety, and it is a complicated task due to the coexistence of various interferences.

Some strategies were previously developed for the direct detection of paraoxon [7,8,9]. However, they are still suffering from one or more drawbacks, such as a time-consuming detection process and expensive and complicated instruments, which cannot meet the requirement for the rapid, straightforward, yet sensitive detection of OPs [10]. Alternatively, coupling cholinesterase inhibition with electrochemical, colorimetric, and fluorescent techniques can detect the OPs residues with a low price and straightforward process [11,12,13]. In particular, fluorometry is more attractive due to its features of high sensitivity, nondestructive detection, and high selectivity [14]. Fluorometry assays usually detect OPs through the quencher-modulated fluorescence of probes, followed by the specific response of the quencher to the hydrolysate of the substrate under the catalysis of cholinesterase. Although rapid and great progress have been achieved, these assays are still hindered by several drawbacks. (1) The fluorescent probes face some limitations, such as toxicity, difficulty to synthesize, and poor stability and dissolution ability in water. (2) In a typical quencher-modulated fluorescence assay, the fluorescence of the probes is quenched by nanomaterial-based energy acceptors, followed by partial/full recovery after introducing the OPs. However, the detection performances are limited both by the quenching efficiency and the degree of fluorescence recovery. Thus, it is highly desired to develop assays for OPs that use low toxic and high-quality fluorescence probes.

Metal nanoclusters (NCs) are a subfamily of fluorescence probes which show excellent fluorescence features, such as relatively strong fluorescence, large Stokes shift, ease synthesis, and good solubility and stability in aqueous solution [15,16,17,18,19,20]. Compared with the widely reported Au and Ag NCs, Cu NCs show additional advantages, such as being abundant in raw materials, cheap in cost, and low in toxicity [21,22,23]. The fluorescence of Cu NCs can also be conveniently tuned by controlling the metal Cu core or the surface ligands [24]. Some groups utilized metal NCs as probes for the detection of OPs. For example, Yan and co-workers designed a fluorometric method for the detection of OPs based on the quenching of the effect of dopaminechrome on Au NCs-tyrosine [25]. Wang et al. reported an assay based on the inner filter effect (IFE) of diaminophenazine to polyvinylpyrrolidone (PVP) protected Cu NCs [26]. Yang’s group reported a ratiometric fluorescent assay for dinotefuran based on the IFE between carbon dots and Cu NCs [27]. Like most of the reported fluorescence assays, these strategies are based on the quenching of Cu NCs, followed by partial fluorescence recovery. The detection sensitivity is limited by the efficiency of fluorescence recovery. In addition, the selectivity of these assays was also hindered by the ease of variation of fluorescence intensity of the quenched Cu NCs by the coexisted interferences [28]. Thus, it is favorable to design a detection pathway by controlling the target recognition and fluorescence output process for sensitive and selective detection of OPs.

**Figure 1:**
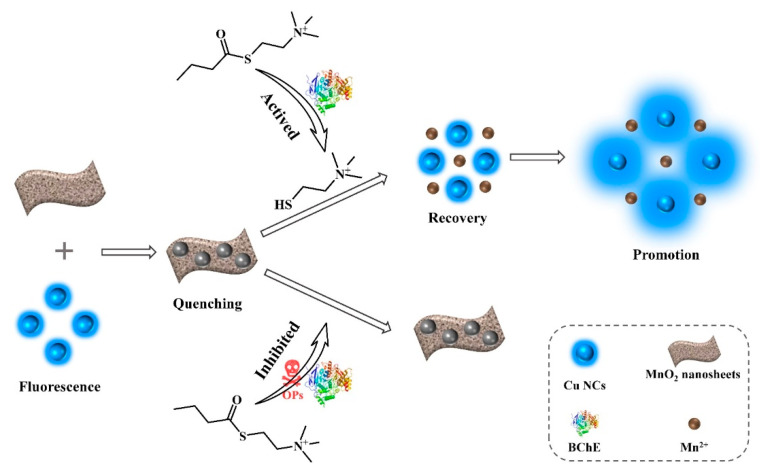
Schematic illustration for the detection of OPs based on the platform of Cu NCs/MnO_2_ nanosheets.

In this work, a platform was developed by constructing a complex of Cu NCs and MnO_2_ nanosheets (Figure 1). The fluorescence of the Cu NCs was effectively quenched by MnO_2_ nanosheets via a dynamic quenching effect. The quenched fluorescence can be fully recovered after introducing butyrylcholinesterase (BChE) and butyrylcholine iodide (BTCh) into the system due to the decomposition reaction between MnO_2_ nanosheets and their hydrolysate. In addition, the fluorescence intensity could be further promoted through the host–guest interactions between Cu NCs and thiocholine. Paraoxon could inhibit the hydrolysate reaction, which leads to the failure of fluorescence recovery and further promotion. The concentration of paraoxon can be evaluated from the change of fluorescence signals in the platform. The assay shows strong tolerance to common interference species and shows promising potential for screen tests of OPs. The practical applications of this assay for the detection of vegetables and fruit were also demonstrated.

## 2. Materials and Methods

### 2.1. Materials

All chemicals, including polyvinyl pyrrolidone (PVP), Co(NO_3_)_2_·6H_2_O, tetramethylammonium hydroxide pentahydrate (TMA·OH·5H_2_O), Al(NO_3_)_3_·9H_2_O and histidine (His), Pb(NO_3_)_2_, lysine (Lys), glucose (Glu), ascorbic acid (AA), hydrogen peroxide (H_2_O_2_, 30 wt%), manganese chloride tetrahydrate (MnCl_2_·4H_2_O), cysteamine (Cys), glycine (Gly), potassium chloride (KCl), calcium chloride dehydrate (CaCl_2_·2H_2_O), sodium chloride (NaCl), copper sulfate pentahydrate (CuSO_4_·5H_2_O) and sodium hydroxide (NaOH) were obtained from Aladdin (Shanghai). 

### 2.2. Apparatus 

The morphology and size of the nanomaterials were observed by transmission electron microscope (FEI, Tecnai G2 F20 S-TWIN, Hillsboro, OR, USA). The fluorescence and UV-vis absorption spectra of the samples were collected through a fluorescence spectrophotometer (HITACHI, F-7000, Tokyo, Japan) and a UV-visible spectrophotometer (Shimadzu, UV-3600, Tokyo, Japan), respectively. The XPS spectra were obtained by an X-ray photoelectron spectrometer (Thermo, ESCALAB-MKII 250, Waltham, MA, USA). 

### 2.3. Preparation of Cu NCs

Cu NCs were synthesized based on our previous work [29]. Typically, 1 g of PVP was added to 20 mL of ultra-pure water, dissolved by ultrasonication for 15 min, and the pH was adjusted to 6.0 using 0.5 M of NaOH solution. Then, 2 mL of AA aqueous solution (0.1 M) and 0.2 mL of CuSO_4_ solution (0.1 M) were added to the above PVP solution and reacted for 6 days under continuous agitation. The as-obtained products were dialyzed against distilled water through a dialysis membrane with a molecular weight cut off of 30,000 for 24 h, and stored at 4 °C for later use.

### 2.4. Preparation of MnO_2_ Nanosheets

MnO_2_ nanosheets were prepared through an ultrasonication-assist top-down method [30]. Typically, 10 mL of MnCl_2_ 4H_2_O (0.3 M) aqueous solution were mixed with 20 mL of TMAOH (0.6 M) solution containing 3 wt% H_2_O_2_ under stirring. The color of the mixture changed from colorless to dark brown, indicating that Mn^2+^ was oxidized to MnO_2_. After stirring for 12 h, the resulting solution was centrifuged (8000 RPM/6 min) for separation. The precipitation was washed with methanol and ultrapure water for five times, respectively. Finally, the precipitate was dried in a vacuum dryer at 60 °C for 6 h. Then, 10 mg of MnO_2_ powder were added to 20 mL ultrapure water and dissolved under ultrasonic conditions for 20 h. The solution was then centrifuged for 30 min, and the supernatant was collected for sequencing use. 

### 2.5. Detection of BChE 

Various concentrations of BChE (20 μL) were mixed with 20 μL of ATCh (12 mM) and 20 μL 10 mM PBS buffer (pH = 7.4, 10 mM), which was allowed to react for 30 min at 37 °C. Then, the mixture was added to the platform containing 10 μL of MnO_2_ nanosheets (26 μL/mL) and 10 μL of Cu NCs, which were allowed to react for 30 min. Finally, the solution was diluted to 200 μL with ultrapure water before the fluorescence spectrum was collected.

### 2.6. Detection of OPs 

In the assay, 20 μL of a solution containing different concentrations of paraoxon was mixed with 20 μL of BChE (1.5 U/mL), allowing it to react for 30 min under 37 °C. Then, 20 μL of ATCh (12 mM) and 20 μL of PBS (pH = 8.5, 10 mM) were added to the above mixture and reacted at 37 °C for 0.5 h, which was injected into the complex of Cu NCs and MnO_2_ nanosheets. The system was allowed to react for 28 h at room temperature, and the solution was diluted to 200 μL with ultrapure water before measuring the fluorescence spectra.

## 3. Results

### 3.1. Characterization of Cu NCs and MnO_2_ Nanosheets

**Figure 2:**
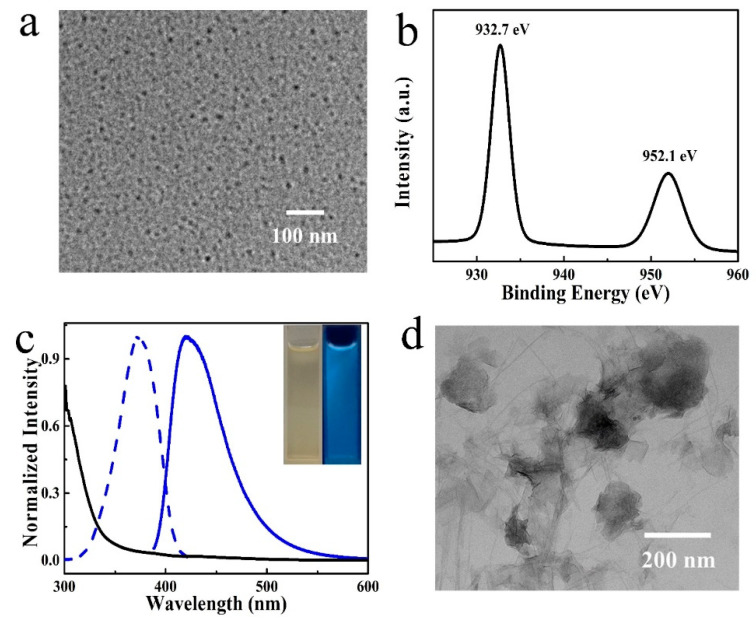
Characterization of Cu NCs and MnO_2_ nanosheets. (**a**) TEM, (**b**) high-resolution Cu 2p XPS spectrum, (**c**) fluorescence emission (blue solid line), excitation (blue dotted line), and UV-visible absorption spectra of Cu NCs. (**d**) High-resolution Mn 2p XPS spectrum of MnO_2_ nanosheets.

Cu NCs were synthesized through a PVP templated chemical reduction method, according to our previous works [29]. The as-obtained products were quasi-spherical in shape with a diameter of around 3 nm, which was well dispersed in the aqueous solution (Figure 2a). The clusters were composed of C, N, O, and a trace amount of Cu, as indicated on the full scan XPS spectrum (Figure S1). The composition of Cu NCs was further studied through FTIR measurement. As shown in Figure S2, an almost identical FTIR spectrum was recorded in the cases of Cu NCs and PVP. The strong band located at 1286 cm^−1^, corresponding to CH_2_ wagging (C-N), suggests the presence of N in the Cu NCs, originating from PVP. The reduction of Cu^2+^ was confirmed by the absence of a characteristic peak around 942 eV in the high-resolution spectra of Cu 2p (Figure 2b). The typical peaks of 932 and 952 eV belong to 2p3/2 and 2p1/2 of Cu atom or Cu^+^, indicating that the valence state is likely between 0 and 1, which is in compliance with previous research [31]. The clusters show intensive blue emission, peaking at 423 nm in the excitation wavelength range from 300 to 400 nm (Figure 2c). No surface plasmon resonance peaks corresponding to Cu nanoparticles were observed on the UV-visible absorption spectra of Cu NCs. Strong absorption bands in the UV range were recorded, attributed to the inter-band electronic transitions of the Cu NCs [32]. The as-obtained Cu NCs were chemical and colloidal stable, with the fact that their fluorescence intensity did not change after 1 month of storage under room temperature.

The MnO_2_ nanosheets were synthesized through an ultrasonication-assisted top-down method [33]. The full scan XPS spectrum of the MnO_2_ nanosheets suggests the presence of C, O, and Mn elements (Figure S3). The high-resolution XPS spectrum of Mn2p (Figure S3) also confirms the formation of MnO_2_, which gives two obvious peaks located at 641.7 and 653.3 eV, corresponding to Mn2p3/2 and Mn2p1/2 binding energies, respectively (Figure S3) [34,35]. After ultrasonication treatment, thin layers of nanosheets could be obtained, as presented in the TEM images of the sample (Figure 2d). 

### 3.2. Modulation of Fluorescence Properties of Cu NCs by MnO_2_ Nanosheets

**Figure 3:**
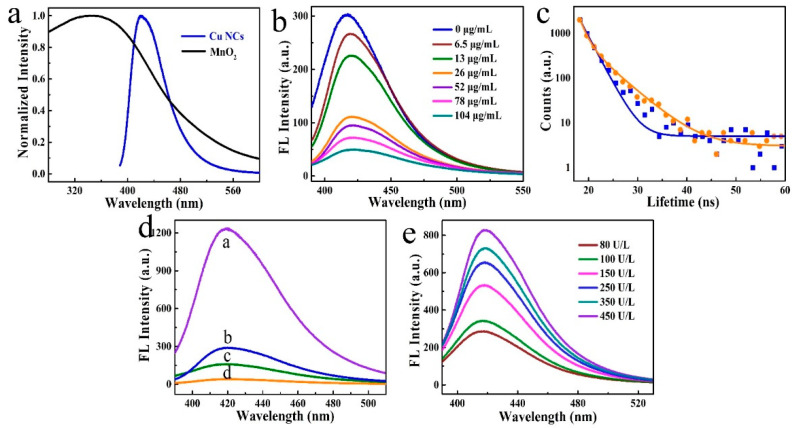
(**a**) Fluorescence emission (blue line) of Cu NCs and UV-visible absorption spectra of MnO_2_ nanosheets; (**b**) Fluorescence spectra of Cu NCs after adding different concentration of MnO_2_ nanosheets; (**c**) Fluorescence decay curves of Cu NCs with (yellow line) and without (blue line) adding MnO_2_ nanosheets; (**d**) Fluorescence spectra of the combination of Cu NCs (**b**), Cu NCs/MnO_2_ nanosheets (**d**), Cu NCs/MnO_2_ nanosheets + BChE (**a**), and Cu NCs/MnO_2_ nanosheets + BChE + OPs (**c**); (**e**) Fluorescence spectra of the detection platform by adding different concentrations of OPs. All the emission spectra were collected under the excitation of 380 nm.

The MnO_2_ nanosheets show a relatively broad absorption band in the wavelength from 300 to 550 nm (Figure 3a), which has a huge overlap (>100 nm) with the fluorescence spectrum of Cu NCs. This suggests the possibility of fluorescence quenching on Cu NCs by MnO_2_ nanosheets after forming a complex [24,30]. Forming a complex reduces the distance between Cu NCs and MnO_2_ nanosheets, and the overlap of fluorescence and absorption suggests the feasibility of energy transfer or light-blocking between these components. As expected, obvious fluorescence quenching was observed upon mixing Cu NCs with MnO_2_ nanosheets (Figure 3b). The quenching efficiency was calculated according to the formula E (%) = (1 – F/F_0_)*100%, where F and F_0_ are the fluorescence intensities of Cu NCs in the presence and absence of MnO_2_ nanosheets, respectively. The quenching efficiency increased with the increasing concentration of MnO_2_ nanosheets, and efficiency of higher than 80% was achieved when 100 μg/mL of the MnO_2_ nanosheets were added (Figure S4). The quenching of luminescent materials by nanomaterials could be explained by a static quenching effect (SQE) or dynamic quenching effect (DQE). In a typical SQE process, a new non-luminescent complex is formed, and the emission lifetime almost remains identical. DQE usually leads to the change in emission lifetime, which involves the deactivation of non-radiative pathways. To obtain more insights into the quenching mechanism, the fluorescence decay curves of Cu NCs with and without adding MnO_2_ nanosheets were recorded (Figure 3c, Table S1). The obvious increase in emission lifetime from 2.8 to 3.6 ns suggests the quenching is a DQE process. The quenching process can also be described by the Stern–Volmer equation (Equation (1)) [36].

F/F_0_ = 1 + K_SV_[Q] (1)

where F and F_0_ is the fluorescence intensity of Cu NCs and their complex with MnO_2_ nanosheets, respectively. [Q] is the concentration of MnO_2_ nanosheets. KSV is a constant for Stern−Volmer quenching, and it can be calculated by the flowing equation (Equation (2)):

(2) $$K_{\mathrm{SV}}=k_{q}\times \tau_{0}$$

where *τ*_0_ is the emission lifetime of the complex of Cu NCs with MnO_2_ nanosheets, and kq is the molecular quenching rate. The type of quenching can be judged by comparing the number of kq with 1 × 10^10^ M^−1^/s. SQE is the dominant process when k_q_ is higher than this number [36]. Instead, it is DQE. The kq of our system was calculated to be 2.1 × 10^3^ M^−1^/s, which suggests the DQE mechanism of our system. 

### 3.3. Feasibility and Mechanism of OPs Detection

The feasibility of OPs detection using the complex of Cu NCs with MnO_2_ nanosheets, combined with the cholinesterase inhibition, was evaluated by studying the fluorescence signal of the system that introduced OPs into the mixture of Cu NCs, MnO_2_ nanosheets, BChE, and OPs (Figure 3d). After adding BChE and BTCh into the complex of Cu NCs and MnO_2_ nanosheets, the fluorescence of the quenched Cu NCs was recovered due to the reduction induced decomposition of the MnO_2_ nanosheets by thiocholine. In addition, the thiocholine further reacted with Cu NCs through the electron donation groups and the surface ligands of Cu NCs, which further promoted fluorescence intensities. Thus, adding BChE and BTCh resulted in the selective fluorescence promotion of quenched Cu NCs, which was about 26 times higher than the quenched Cu NCs and 3 times higher than the initial intensity of Cu NCs (Figure 3d). We attributed the huge increase of fluorescence intensity to two processes, namely the decomposition of the MnO_2_ nanosheets and further promotion through surface treatment. Thiocholine, the product of BTCh under the catalysis of BChE, reacted with the MnO_2_ nanosheets through a redox and reduction reaction between the thiol groups and Mn^4+^. This induced the decomposition of the MnO_2_ nanosheets, which resulted in the fluorescence recovery of the quenched Cu NCs. The electron-rich groups on the thiocholine further reacted with the surface of the Cu NCs, leading to the promotion of fluorescence intensity, which was consistent with previous works [30].

OPs, selecting paraoxon as a proof of concept, can effectively inhibit the catalyst of BChE towards BTCh, which results in the failure of fluorescence promotion. Thus, the change in the fluorescence intensity can reflect the presence of OPs. To obtain the best detection performance, the conditions, including the pH of the system, and the reaction time, were optimized, and the ratio between the fluorescence intensity with (F) and without (F_0_) adding OPs was used to evaluate these conditions. The best ratio was achieved when the pH was 3.0 and the reaction time was 28 h (Figure S5). 

### 3.4. Quantitative Detection of BChE and OPs

Under the optimized conditions, the complex of Cu NCs was challenged to the quantitative detection of OPs. As shown in Figure 3e, with the increase in BChE concentration, the fluorescence intensity of Cu NCs increased, and a fine linear dependence between BChE concentration and F/F_0_ was observed in the range from 80 to 450 U/L (Figure S6). A linear regression equation of F/F_0_ = 20.5 lg[BChE]–29.5 (Figure S6) was fitted, with an R^2^ of 0.993. A limit of detection (LOD) of 27.5 U/L was calculated. This number was one of the highest detection performances among the recently reported works, as shown in Table 1.

**Figure 4:**
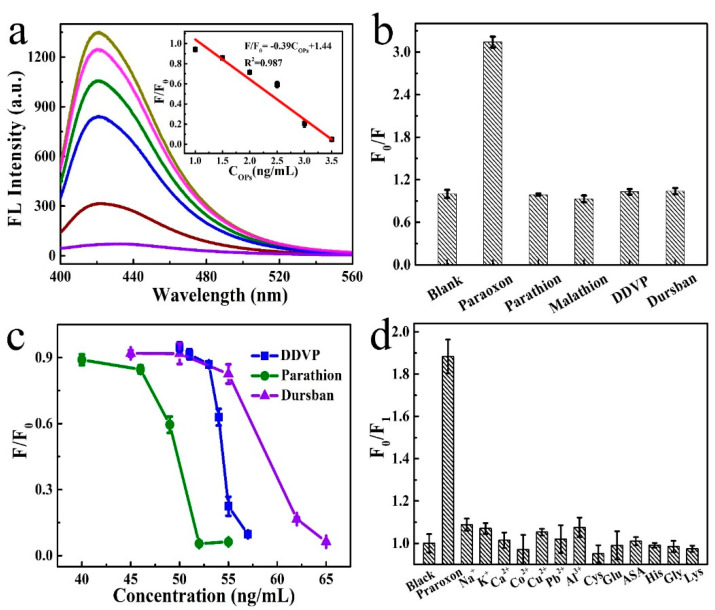
(**a**) Fluorescence emission spectra of the detection platform after adding different concentrations of paraoxon. Relationship between the ratio of fluorescence intensities and the concentrations of paraoxon showing as inset; (**b**) ratio of fluorescence intensities of the detection platform by adding different types of OPs; (**c**) evolution of the ratio of fluorescence intensities of the platform as a function of OPs concentrations; (**d**) selectivity test of the detection platform against different interference species, as presented on the frame. All the fluorescence intensities were recorded at 423 nm, under the excitation of 380 nm.

Due to the excellent detection performance of the complexes, their applications in OPs detection were further evaluated. Paraoxon, as a proof of concept, was introduced into the system, which delayed the enzymatic reactions. This resulted in the failure of fluorescence recovery and further promotion of Cu NCs and different fluorescence intensities were recorded by adding different concentrations of paraoxon. Gradually decreased fluorescence was observed, with the increase of the concentration of paraoxon, as shown in Figure 4a. A fine linear dependence between paraoxon concentration and FP/FP0 was observed in the range from 1.0 to 3.5 ng/mL, giving a linear regression equation of F/F_0_ = −0.39COP_S_ + 1.44, with an R^2^ of 0.987 (Figure 4a). A LOD as low as 0.22 ng/mL was calculated. The detection performances were compared with recently reported works (Table 2). These numbers are among the best detection performances and are comparable with these assays using carbon dots and COF as fluorescence probes. 

To explore the possibility of a screening test of other OPs, we studied the sensing platform for the other three types of pesticides, namely dichlorvos, parathion, and Dursban. Almost no fluorescence response was observed by adding these OPs in a concentration lower than 40 ng/mL, with a fluorescence ratio kept at 1.0 (Figure 4b). In contrast, obvious differences were observed in the cases of adding the same concentration of paraoxon (Figure 4b). This suggests the potential for screen tests of OPs. After increasing the concentrations of these OPs, response signals appeared at 50–57 ng/mL, 40–52 ng/mL, and 45–65 ng/mL for dichlorvos, parathion, and Dursban, respectively (Figure 4c).

### 3.5. Selectivity and Practical Applications

Selectivity is another important characteristic for evaluating the performance of an assay. Thus, the impacts of some common electrolytes and biological species, including Na^+^, K^+^, Ca^2+^, Co^2+^, Cu^2+^, Pb^2+^, Al^3+^, Cys, Glu, ASA, His, Gly, and Lys, were investigated (the concentration of each substance is shown in Table S2). As shown in Figure 4d, an obvious fluorescence response was observed when adding paraoxon into the detection system. In contrast, almost the same fluorescence signal was recorded with that of the blank sample for the cases of Ca^2+^, Cu^2+^, Pd^2+^, Glu, His, Gly, and Lys. The signals for Co^2+^ and Cys were slightly lower than that of the blank sample. While a slight increase in the fluorescence signal was observed (less than 10%) in the cases of Na^+^, K^+^, and Al^3+^, due to the interference of electrolytes. These results indicate the outstanding selectivity of our assay. In addition, we also studied the salt tolerance of the assay by testing the detection performance under different NaCl concentrations. As shown in Figure S7, only an extremely small fluctuation could be observed under different concentrations of NaCl in the range from 0 to 100 nM, which indicates the high robustness of the assay.

Given the high sensitivity and selectivity of our assay, it was further challenged to detect OPs in agricultural samples, and the commonly consumed apples, cowpeas, and pakchoi were selected. After routine treatment, these samples were detected directly, and the concentration of paraoxon was calculated using the calibration curve shown in Figure 4a. The concentration of paraoxon was 10.9, 7.9, and 4.4 ng for apples, cowpeas, and pakchoi, respectively. These numbers are consistent with the normal concentration range of pesticide residues and reported results [37,38]. The accuracy of the assay was further evaluated by standard addition experiments in real samples. Obvious changes in fluorescence signals were recorded with an extremely minor change in the concentration of paraoxon (10, 20, and 30 ng/mL), and acceptable recoveries ranging from 94.2% to 107.9% (Table 3) were obtained, which further confirms the reliability of the assay.

## 4. Discussion

Cu NCs were integrated with MnO_2_ nanosheets, forming a detection platform to modulate the fluorescence of Cu NCs. The fluorescence was effectively quenched through a dynamic quenching effect by MnO_2_ nanosheets. OPs modulated the hydrolysate reaction of BChE and BTCh, which further controlled the decomposition of MnO_2_ nanosheets and host–guest interactions between Cu NCs and thiocholine. This resulted in differences in the fluorescence intensity by adding different concentrations of OPs, and a LOD as low as 0.22 ng/mL was achieved. The outstanding tolerance to common interference species endows the platform for the detection of OPs in real samples. Our results provide new insights into the functionalization and modulation of the fluorescence properties of Cu NCs, which will facilitate their wide applications in nanomedicine and environmental science.

## Figures and Tables

**Table 1:** Comparison of the detection performances of our assay for BChE with recently reported works.

Detecting Materials Used	Linear Range (U/L)	LOD (U/L)	Reference
MnO_2_-NSs -PDA	50–450	40.65	Microchem. J., 2021, 166, 106.
IPAN	25,000–31,000	11.7	Anal. Chem., 2020, 92, 13405.
3D printing device	1000–12,000	100	Sens. Actuators B., 2018, 258, 1015.
CDs-OPD	1000–25,000	400	Biosens. Bioelectron., 2022, 196, 113691
3D ER-PS	80–550	30.3	Anal. Chem., 2019, 20, 12874.
Cu NCs-MnO_2_ NS	80–450	27.5	This work

**Table 2:** Comparison of the detection performances of our assay for OPs with recently reported works.

Detecting Materials Used	Linear Range (ng/mL)	LOD (ng/mL)	Reference
[Fe(CN)_6_]^3−^	0–30	5	Anal. Chim. Acta, 2019, 1060, 97.
COF_ML-DHTA_	0.57–300	0.19	Anal. Methods, 2021, 13,5727.
AuNPs-MoS_2_-rGO/PI	5–150	1.4	Bioelectrochemistry, 2020, 131, 107392.
DTNB-CDs	1–1000	0.4	Sens. Actuators B., 2018, 260, 563.
Su-TPE/PrS	0–384	5.28	J. Mol. Liq., 2021, 333, 115980.
MnO_2_ NS-Cu NCs	1.0–3.5	0.22	This work

**Table 3:** Recovery test results of OPs in different samples using our assay.

Sample	Spiked (ng/mL)	Detection (ng/mL)	Recovery (%)	RSD (%)
Apple	10	9.9	99.0	0.383
	20	21.3	106.5	0.396
	30	29.3	97.6	0.750
Cowpeas	10	10.1	101.0	0.175
	20	19.7	98.5	0.647
	30	28.3	94.3	0.575
Pakchoi	10	9.5	95.0	0.360
	20	19.6	98.0	0.354
	30	32.4	108.0	0.377

## Data Availability

Not applicable.

## Supplementary Materials

The following supporting information can be downloaded at: https://www.mdpi.com/article/10.3390/nano12091429/s1, Figure S1: Full scan XPS spectrum of Cu NCs; Figure S1: FTIR spectra of PVP and Cu NCs; Figure S3: Full scan XPS spectrum of MnO_2_ nanosheets; Figure S4: Evolution of quenching efficiency as a function of MnO_2_ nanosheets concentrations; Figure S5: Evolution of ratio of fluorescence intensities as a function of pH and reaction time; Figure S6: Relationship between the ratio of fluorescence intensities and the concentration of BChE; Figure S7. Evolution of fluorescence intensity ratio as a function of NaCl concentration. Table S1: Fitted results of fluorescence decay curves of Cu NCs with and without adding MnO_2_ nanosheets; Table S2: Concentrations of the interference species.
