# Supplementary material for: Highly Selective Detection of Paraoxon in Food Based on the Platform of Cu Nanocluster/MnO2 Nanosheets
Source: Nanomaterials (Basel). 2022 Apr 22;12(9):1429. doi: 10.3390/nano12091429 (PMC9100620; doi:10.3390/nano12091429)
Supplement: Supplementary file 1 [file nanomaterials-12-01429-s001.zip › nanomaterials-1659627-supplementary.pdf]

Highly Selective Detection of Organophosphorus Pesticides in Food Based on the Platform of Cu Nanocluster/MnO_2 Nanosheets

Shuo Liu [1,†], Peng Zhang [2,†], Yuling Miao [1], Chenmin Li [1], Yu-e Shi [1,*], Jinhua Liu [3], Yun-kai Lv [1] and Zhenguang Wang[1,*]

[1] Key Laboratory of Chemical Biology of Hebei Province; Key Laboratory of Medicinal Chemistry and Molecular Diagnosis, Ministry of Education; College of Chemistry & Environmental Science, Hebei University, Baoding 071002, China
[2] Department of Pharmacy, Shenzhen Luohu People's Hospital, No. 47 Youyi Rd, Luohu, Shenzhen 518001, China
[3] Institute of Advanced Materials (IAM) , Key Laboratory of Flexible Electronics (KLOFE), Nanjing Tech University, 30 South Puzhu Road, Nanjing 211816,
* Correspondence: zgwang@hbu.edu.cn, shiyue1220@163.com
† These authors contributed equally to this work.

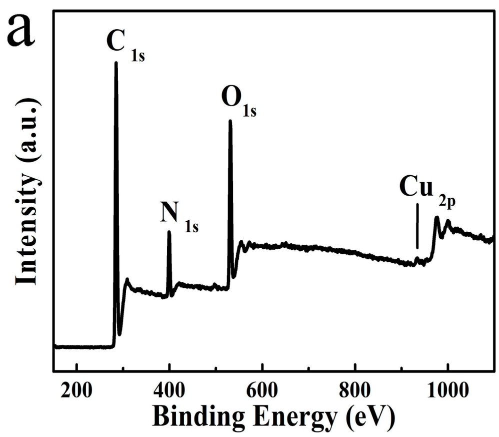

Figure S1. Full scan XPS spectrum of CuNCs.

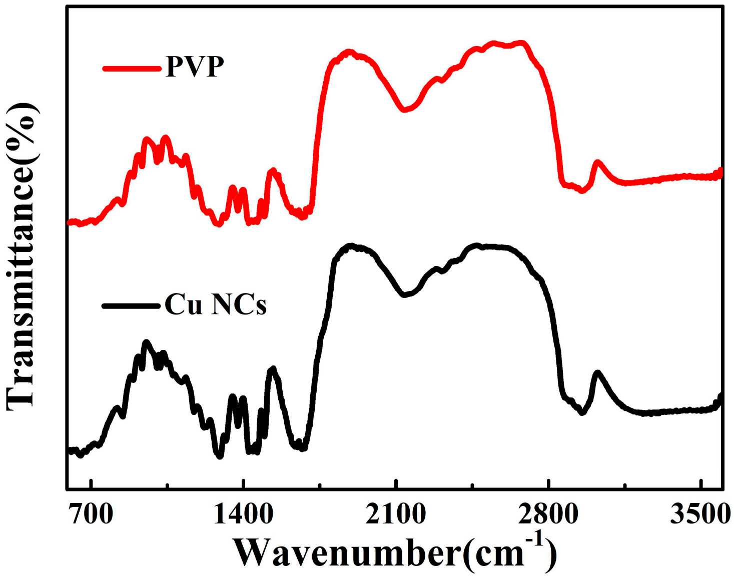

Figure S2. FTIR spectra of PVP (red line) and Cu NCs (black line).

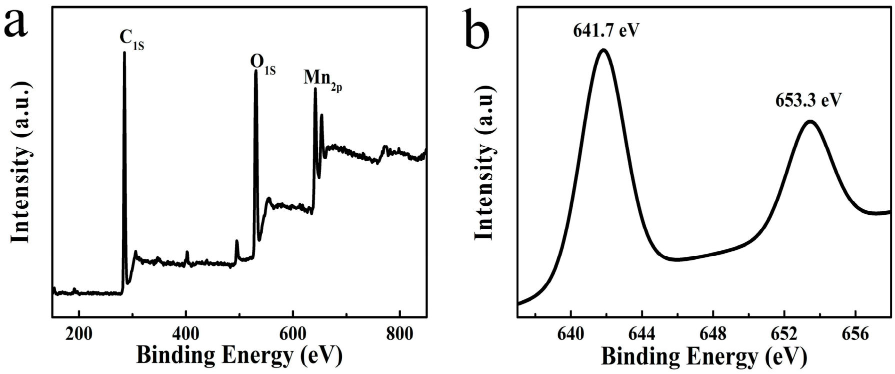

Figure S3. (a) Full scan XPS spectrum of MnO_2 nanosheets; (b) High resolution Mn 2p XPS spectrum of MnO_2 nanosheets.

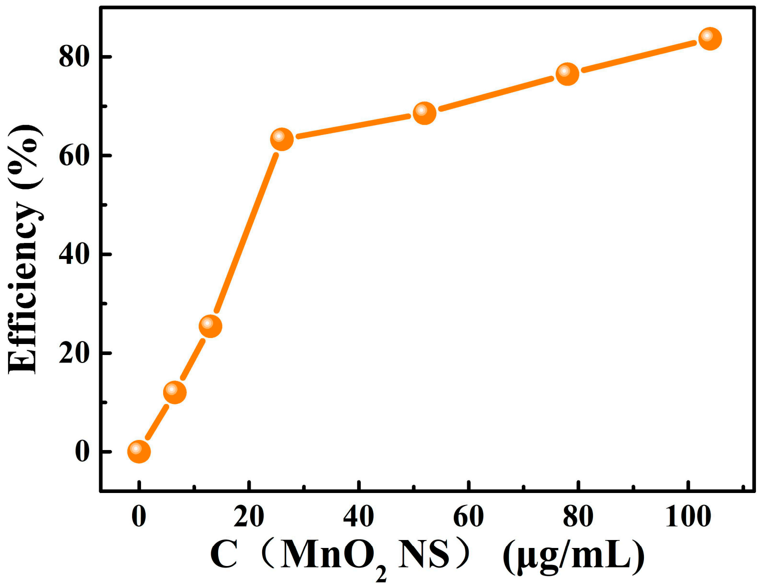

Figure S4. Evolution of quenching efficiency as a function of MnO_2 nanosheets concentrations.

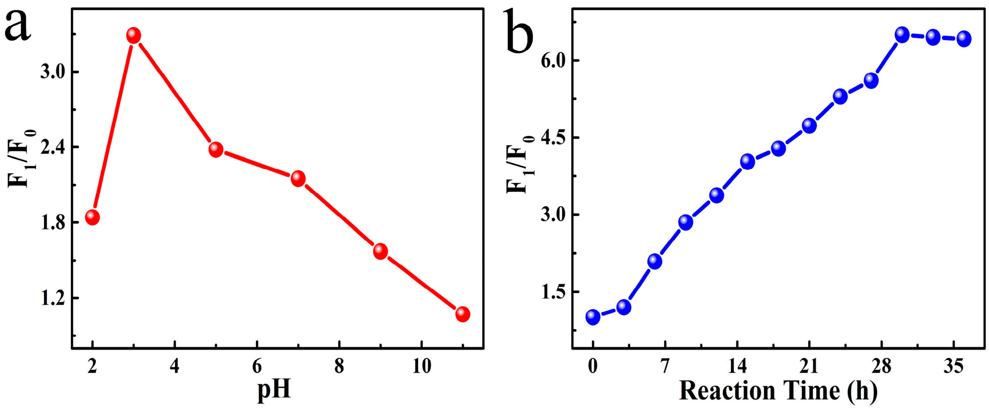

Figure S5. Evolution of ratio of fluorescence intensities as a function of pH (a) and reaction time (b). All the intensities were recorded at 423 nm, excited at 380 nm.

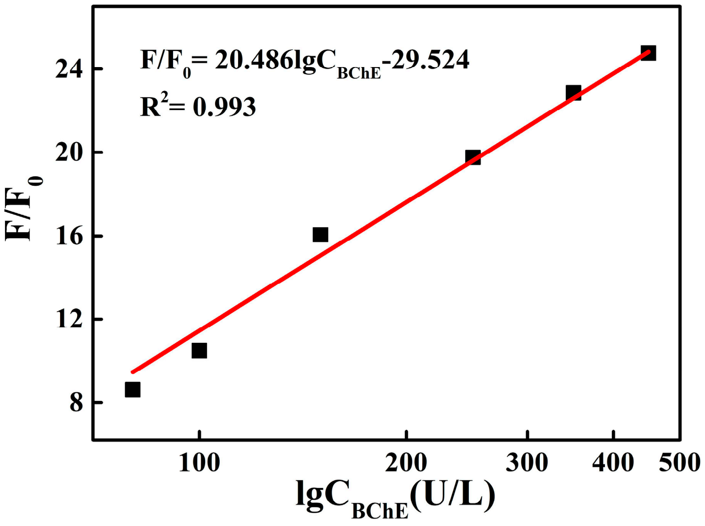

Figure S6. Relationship between the ratio of fluorescence intensities and the concentration of BChE. All the intensities were recorded at 423 nm, excited at 380 nm.

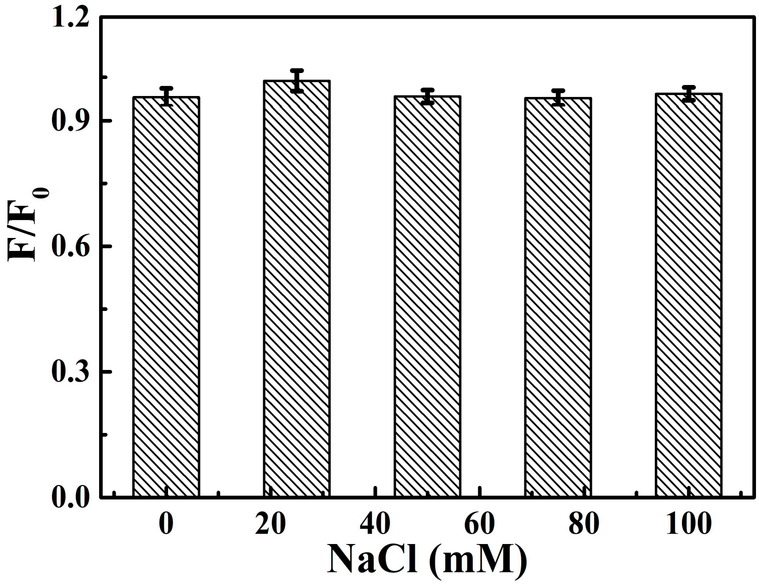

Figure S7. Evolution of fluorescence intensity ratio as a function of NaCl concentration.

Table S1. Fluorescence lifetimes (τ_{1-3}, ns) and fractions of the emission intensity (f_{1-3},%) obtained from the fitting of experimental fluorescence decay data by three-exponential functions of Cu NCs with and without adding MnO_2 nanosheets, from which the average emission lifetimes (τ_{avg}, ns) were calculated. All the curves were detected at 423 nm, under the excitation of 380 nm.

Sample	τ_1（ns）	τ_2（ns）	τ_3（ns）	$\tau_{average}$（ns）
Cu NCs	1.49(42.59%)	2.82(51.11%)	11.06(6.30%)	2.78
Cu NCs +MnO_2 nanosheets	0.92(23.08%)	3.40(65.40%)	10.30(11.53%)	3.62

Table S2. Concentrations of the interference species.

Interference	Concentration
Na^+	1.6 mM
K^+	2.0 mM
Ca^{2+}	14 mM
Co^{2+}	1.0 mM
Cu^{2+}	1.0 mM
Pb^{2+}	1.0 mM
Al^{3+}	1.0 mM
Cysteine (Cys)	250 μM
Glucose (Glu)	6 mM
Vitamine (ASA)	50 μM
Histidine (His)	50 μM
Glycine (Gly)	50 μM
Lysine (Lys)	50 μM
